# The Role of Neoadjuvant Immunotherapy in the Management of High-Risk Stage III Resectable Melanoma: A Literature Review

**DOI:** 10.3390/cancers17132152

**Published:** 2025-06-26

**Authors:** Jobran Mansour, Cecelia E. Schmalbach

**Affiliations:** Department of Otolaryngology-Head and Neck Surgery, Lewis Katz School of Medicine at Temple University, Philadelphia, PA 19140, USA; cecelia.schmalbach@tuhs.temple.edu

**Keywords:** neoadjuvant immunotherapy, cutaneous melanoma, adjuvant immune-checkpoint inhibitors

## Abstract

Neoadjuvant immunotherapy is changing the treatment paradigm for patients with advanced cutaneous melanoma. This systemic approach leverages the presence of tumor antigens to generate a robust and durable antitumor immune response compared to traditional adjuvant therapy. The comprehensive review highlights contemporary clinical trials shaping the landscape of melanoma treatment algorithms. Single-modality agents and combination regimens are outlined along with response-adapted strategies, which aim to balance efficacy and toxicity. These novel neoadjuvant immunotherapy strategies promise to further refine treatment in a personalized manner, ideally offering patients the opportunity for greater response, reduced treatment toxicity, and improved long-term survival.

## Simple Summary

Multiple trials have established neoadjuvant immunotherapy as a promising approach for resectable stage III melanoma, showing improved pathological response and event-free survival compared to adjuvant therapy. Trials such as OpACIN, OpACIN-neo, SWOG S1801, PRADO, and NADINA support early immune engagement and response-adapted treatment strategies. Optimal dosing of ipilimumab plus nivolumab balances efficacy with tolerability, while relatlimab-nivolumab and T-VEC combinations offer additional options. These findings support personalized neoadjuvant strategies that enhance survival, reduce morbidity, and improve quality of life in high-risk melanoma patients.

## 1. Introduction

Neoadjuvant therapy has been a cornerstone of cancer management for decades, primarily aimed at optimizing treatment outcomes prior to surgical intervention. This approach focuses on reducing tumor burden to facilitate complete resection, minimize surgical complications, and enhance postoperative function and quality of life [1]. It is particularly advantageous in cancers where organ preservation is critical or where surgery poses significant morbidity to form and function, such as breast, gastroesophageal, head and neck (HN), and anorectal cancers. Beyond tumor reduction, neoadjuvant therapy provides a unique opportunity to assess tumor response to treatment, enabling early, personalized identification of resistant disease and informing subsequent therapeutic decisions. This review highlights landmark neoadjuvant trials that reshaped the treatment of cutaneous melanoma and discusses ongoing studies in the field.

## 2. Melanoma Immunotherapy Overview

Immunotherapy (IO) revolutionized the treatment landscape for advanced, unresectable melanoma, offering improved survival outcomes for patients [2]. PD-1 checkpoint inhibitors such as nivolumab and pembrolizumab, alone or in conjunction with anti-CTLA-4 agents, demonstrated significant efficacy in enhancing the body’s immune response against melanoma cells [3]. Landmark trials such as Keynote-006 and CheckMate-067 demonstrate ongoing survival benefits of anti-PD-1 agents among advanced-stage melanoma patients with improved overall survival (OS) and progression-free survival (PFS) [3,4]. These therapies transformed melanoma management and are now integral in the treatment of metastatic melanoma, providing durable responses and extending patient survival [5]. Although melanoma remains one of the deadliest cancers, accounting for approximately 59,000 deaths worldwide [6], IO improved 5-year OS rates in stage IV melanoma from 15% to 52% with combination anti-PDL-1 and anti-CTLA-4 therapy [3,4,7,8,9].

Building upon the success in stage IV unresectable melanoma, IO was incorporated into adjuvant treatment protocols for patients with resected stage III and IV melanoma. The postoperative administration of agents such as pembrolizumab aims to eradicate residual microscopic disease, thereby reducing the risk of recurrence. Clinical trials demonstrated that adjuvant IO significantly prolongs recurrence-free survival (RFS) in high-risk melanoma. Important clinical trials such as Keynote-054 and CheckMate-238 demonstrated prolonged survival and PFS benefit with adjuvant IO in the setting of advanced-stage melanoma, with 5-year OS reaching 76% [10,11]. Table 1 provides of summary of neoadjuvant IO trials for resectable stage III/IV melanoma.

## 3. Rational for Neoadjuvant Immunotherapy in Resectable Stage III Melanoma

**Figure 1:**
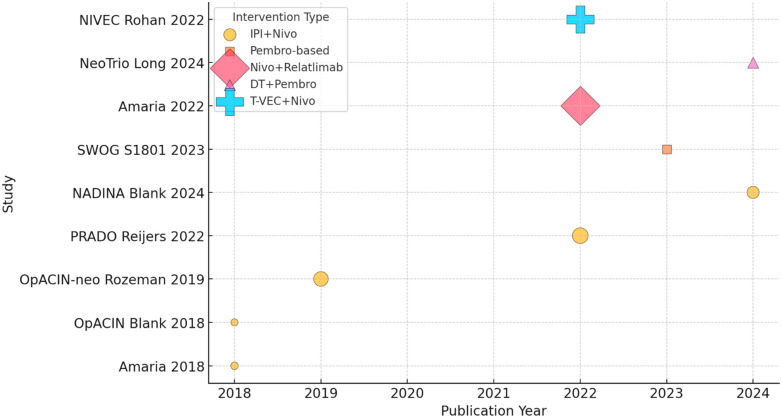
Timeline of neoadjuvant immunotherapy trials in melanoma (shape-coded intervention, bubble size based on N patients) [12,15,16,17,18,19,20,21,22].

The rationale behind neoadjuvant IO in stage III melanoma is based on its potential to enhance systemic antitumor immunity, improve surgical outcomes, and reduce recurrence rates. Administering immune checkpoint inhibitors (ICIs) such as anti-PD-1 (nivolumab, pembrolizumab) or combined anti-PD-1/CTLA-4 (nivolumab/ipilimumab) in the neoadjuvant settings allows early activation of tumor-specific T cells while the tumor is still present, providing a broader repertoire of antigens to stimulate a more robust and durable immune response [12]. Studies have shown that neoadjuvant IO can downstage tumors, making them more amenable to complete surgical resection and potentially reducing the need for extensive lymphadenectomy, which is associated with significant morbidity [13]. Additionally, neoadjuvant therapy offers an opportunity to assess treatment response before surgery, allowing for early identification of patients who may require escalated postoperative therapy [14]. Pathologic response rates have been correlated with improved RFS and OS, further supporting the effectiveness of this approach. As ongoing clinical trials refine neoadjuvant strategies, this approach is emerging as a transformative treatment paradigm for patients with stage III melanoma (Figure 1).

## 4. Neoadjuvant Immunotherapy in Resectable Stage III Melanoma: Available Data and Key Studies

### 4.1. Nivolumab vs. Ipilimumab + Nivolumab in Resectable Clinical Stage III or Oligometastatic Stage IV Disease [12]

Based on preclinical data of neoadjuvant efficacy, Amaria et al. [12] designed a phase II randomized study to test the feasibility, efficacy, and immune correlates of single checkpoint inhibitor (Nivolumab) vs. combination checkpoint inhibitors (Ipilimumab 3 mg/kg plus Nivolumab 1 mg/kg) every 3 weeks for up to three doses followed by surgery and optional adjuvant therapy. The results showed that monotherapy was associated with lower radiological response (25% vs. 75%, *p* = 0.0339) and pathologic complete response (pCR, no viable tumor) (25% vs. 45%, *p* = 0.40) when compared to combination therapy. While pCR improved in the combination arm, it did not reach statistical significance. Expectedly, combination therapy was associated with significantly worse grade 3+ immune-related adverse effects (irAEs), 73% vs. 8%, respectively. Amaria et al. proved that combination ipilimumab + nivolumab demonstrated higher response and pCR rates, but at the cost of significantly greater toxicity with the current combination regimen.

### 4.2. OpACIN Trial: Neoadjuvant Ipilimumab + Nivolumab vs. Adjuvant Nivolumab Alone in Resectable Stage III Melanoma [15]

In the OpACIN trial, Blank et al. [15] conducted a phase Ib trial to assess the efficacy and safety of neoadjuvant combination ICI. The study included 20 patients with palpable stage III melanoma to receive ipilimumab (3 mg/kg) and nivolumab (1 mg/kg) as either two courses before surgery and two courses post-surgery (neoadjuvant arm) or four courses after surgery (adjuvant arm). Among nine patients treated in the neoadjuvant arm, seven (78%) had a pathological response, three had pCR, three had near-complete pathological response (near pCR, ≤10% viable tumor), and one had partial response (pPR, >10–≤ 50% viable tumor). No patients experienced relapse after a follow-up of 25.6 months. All patients were able to undergo surgery at the preplanned time. However, grade 3/4 irAEs occurred in 90% of patients in both arms without a significant difference between the two groups, indicating high toxicity with the current dosing regimen. Due to toxicity, only 1 out of 10 patients in each arm completed all four treatment cycles. Neoadjuvant therapy was associated with greater expansion of tumor-resident T cell clones in the peripheral blood. The OpACIN trial proved that neoadjuvant ipilimumab + nivolumab is immunologically and clinically promising, with high pathological response rates and potentially better RFS; however, toxicity remains a major challenge, warranting future development of less intense regimens.

### 4.3. OpACIN-Neo Trial: Optimal Combination Dosing Schedule of Neoadjuvant Ipilimumab Plus Nivolumab in Macroscopic Stage III Melanoma [16]

Rozeman et al. expanded on previous work (OpACIN trial [15]) in a phase II study to investigate the optimal combination dosing of neoadjuvant ipilimumab plus nivolumab in macroscopic stage III melanoma. Patients were randomized to one of three neoadjuvant dosing schedules: group A: two cycles of ipilimumab (3 mg/kg) plus nivolumab (1 mg/kg) every 3 weeks; group B: two cycles of ipilimumab (1 mg/kg) plus nivolumab (3 mg/kg) every 3 weeks; and group C: sequential ipilimumab (3 mg/kg) every 3 weeks followed by two cycles of nivolumab (3 mg/kg) every two weeks. Eighty-six patients were included. Pathological responses occurred in 24 (80%) patients in group A, 23 (77%) in group B, and 17 (65%) in group C. Event-free survival (EFS) and RFS were similar between the groups. Pathological response led to a durable response. None of the 64 patients achieving a pathological response relapsed, compared to 9 of 21 (43%) patients with pathological no response (pNR) who relapsed.

Grade 3–4 irAEs were observed 40% of patients in group A, 20% of patients in group B, and 50% of patients in group C.

High IFN-γ gene expression signature and high tumor mutational burden (TMB) in pretreatment tumor biopsies were associated with a higher rate of pathological response and a lower risk of relapse, suggesting the potential as a predictive biomarker for neoadjuvant IO response. Specifically, the pathologic response rate was 100% vs. 37% in patients with a high TMB/high IFN-γ-related gene expression score versus low TMB/low IFN-γ-related gene expression, respectively. In contrast, PD-L1 expression was not correlated with treatment response. The study concluded that the treatment regimen in group B was the best tolerated dosing schedule of neoadjuvant ipilimumab and nivolumab while still inducing a high pathological response rate. Group B’s regimen is now utilized in neoadjuvant trials including the PRADO trial (see below) [17]. Lastly, long-term follow-up of both the OpACIN and OpACIN-neo trials reported a durable response among patients with pathological response; 2-year estimated RFS was 84% for all patients, 97% for patients achieving a pathological response, and 36% for non-responders [13].

### 4.4. Southwest Oncology Group (SWOG) S1801 Study: Neoadjuvant–Adjuvant Pembrolizumab vs. Adjuvant-Only Pembrolizumab [18]

Patel et al. [18] designed this phase II trial to compare neoadjuvant–adjuvant pembrolizumab versus adjuvant pembrolizumab in patients with resectable stage IIIB–IV melanoma. In total, 313 patients were divided into the neoadjuvant–adjuvant group (three doses of neoadjuvant pembrolizumab, surgery, and fifteen doses of adjuvant pembrolizumab) or the adjuvant group (surgery followed by pembrolizumab (200 mg intravenously every 3 weeks for a total of 18 doses)). Treatment continued for approximately 1 year or until disease recurred or major irAEs developed. The study’s primary endpoint was EFS, including recurrence, disease progression, and irAEs before the initiation of adjuvant therapy. A pCR was observed in 21% of patients who received neoadjuvant therapy, and the radiologic objective response rate was 47%. Among the neoadjuvant group, 12 of 144 patients (8.3%) did not undergo surgery as planned due to disease progression. The study demonstrated a significant improvement in EFS in the neoadjuvant–adjuvant group, with a 2-year EFS rate of 72% compared to 49% in the adjuvant-only group (*p* = 0.004). The treatment was expectedly well tolerated and less toxic than combination neoadjuvant therapy, with grade 3 or higher irAEs occurring in 12% of patients in the neoadjuvant–adjuvant group and 14% in the adjuvant-only group. The incidence of surgery-related adverse events was similar between treatment arms. The addition of neoadjuvant therapy did not increase surgical complications, supporting the feasibility and clinical benefit of neoadjuvant PD-1 blockade in resectable advanced melanoma.

### 4.5. The PRADO Trial: Personalized Response-Directed Surgery and Adjuvant Therapy After Neoadjuvant Ipilimumab and Nivolumab in High-Risk Stage III Melanoma [17]

The PRADO trial is an extension of the OpACIN and OpACIN-neo trials. While therapeutic lymph node dissection (TLND) demonstrated improved local recurrence in locally metastatic patients, it is associated with significant morbidity, such as lymphedema, wound infection, and impaired mobility [23]. Therefore, the PRADO trial investigated whether treatment intensity could be tailored based on index lymph node (ILN) response with de-escalation of therapy (omitting of TLND/adjuvant therapy) for major responders and escalation of therapy (addition of systemic therapy ± radiotherapy) for non-responders. Ninety-nine patients with clinical stage IIIB–D melanoma received neoadjuvant ipilimumab 1 mg/kg plus nivolumab 3 mg/kg for two cycles, followed by resection of a pre-marked ILN, usually the largest metastatic lymph node, to guide subsequent treatment. Patients achieving major pathological response (less than 10% viable tumor, MPR) in the ILN avoided TLND and adjuvant therapy, while those with pPR received TLND alone, and patients with no pathological response underwent TLND with adjuvant therapy (systemic therapy ± radiotherapy). The overall response was 72%, including 61% with MPR (49% pCR; 12% had near pCR). Among sixty patients achieving MPR, 98% avoided TLND and adjuvant therapy. At 24 months, RFS and distant metastasis-free survival (DMFS) rates in MPR patients were 93% and 98%, respectively. The 2-year RFS and DFMS were 64% and 64%, respectively, among patients with pPR, and 71% and 76%, respectively, among non-responders. Grade 3–4 irAEs occurred in 22% of patients within the first 12 weeks. Omission of TLND in patients with MPR was associated with significantly lower surgical morbidity and improved quality of life. The PRADO trial demonstrated that pathologic response assessment via ILN resection is a safe and effective strategy to guide personalized treatment in stage III melanoma. Omission of TLND and adjuvant therapy in patients with major responses led to excellent survival outcomes, reduced morbidity, and improved QoL, supporting treatment de-escalation for responders and intensification for non-responders.

### 4.6. NADINA Trial: Neoadjuvant Nivolumab and Ipilimumab in Resectable Stage III Melanoma [19]

Similar to the PRADO trial, the NADINA trial investigated the possibility of personalized adjuvant therapy based on pathological response following short neoadjuvant therapy. In this phase III trial, 423 patients with macroscopic stage III melanoma were randomized to two cycles of neoadjuvant ipilimumab 80 mg plus nivolumab 240 mg every three weeks, followed by TLND and response-adapted adjuvant therapy (neoadjuvant group), or surgery followed by 12 cycles of adjuvant nivolumab (standard of care; adjuvant group). Patients with an MPR did not receive any adjuvant treatment, and patients who had a pPR or pNR received adjuvant dabrafenib (150 mg twice daily) plus trametinib (2 mg once daily) for 46 weeks if they had BRAF-mutated melanoma or an additional 11 cycles of adjuvant nivolumab (480 mg every 4 weeks) if they had BRAF wild-type melanoma. At a median follow-up of 9.9 months, the 12-month EFS was significantly higher in the neoadjuvant group compared to the adjuvant group (83.7% vs. 57.2%; hazard ratio (HR) for progression, recurrence, or death: 0.32; *p* < 0.001). In the neoadjuvant group, 59.0% of patients had an MPR, 8.0% had a pPR, and 26.4% had pNR. Only 2.4% had progression before surgery. An MPR was associated with a 12-month RFS of 95.1%, while pPR and pNR were associated with a rate of 76.1% and 57.0%, respectively. IrAEs of grade 3 or higher occurred more frequently in the neoadjuvant group (29.7% vs. 14.7%) but were consistent with prior safety profiles of combination ICIs as seen in the OpACIN-neo trial and others [16].

### 4.7. Neoadjuvant Relatlimab and Nivolumab in Resectable Melanoma [20]

This phase II trial explored whether dual ICI, targeting both LAG-3 (lymphocyte-activation gene 3) and PD-1, could provide improved clinical benefit over PD-1 inhibition alone in patients with resectable clinical stage III or oligometastatic stage IV melanoma. Thirty patients receive two cycles of a fixed-dose combination of relatlimab (anti–LAG–3, 160 mg) plus nivolumab (480 mg) administered every 4 weeks, surgery at week 9, and then adjuvant therapy with the same combination for an additional 10 cycles. Among the 30 treated patients, the pCR was 57%, and 70% achieved some degree of pathologic response (≤50% viable tumor). The radiographic response rate was 57%, though discordance between imaging and pathology was noted. At a median follow-up of 24.4 months, the RFS was 92% for patients with any pathologic response compared to 55% for those without (*p* = 0.005). No grade 3–4 irAEs occurred during neoadjuvant therapy; however, 26% of patients experienced grade ≥3 irAEs during the adjuvant phase, and 33% discontinued adjuvant treatment due to toxicity. These findings demonstrate that neoadjuvant relatlimab plus nivolumab is a highly active and well-tolerated combination, achieving deep pathological responses and durable clinical outcomes in resectable high-risk stage III and oligometastatic stage IV melanoma.

### 4.8. NeoTrio Trial: Neoadjuvant Pembrolizumab, Dabrafenib and Trametinib in BRAFV600-Mutant Resectable Melanoma [21]

Long-term results from the NeoCombi trial demonstrated that neoadjuvant targeted therapy in stage IIIB-C melanoma has limited advantage over adjuvant therapy due to a high rate of recurrence [24]. Nonetheless, this phase II NeoTrio trial by Long et al. [21] aimed to investigate whether neoadjuvant targeted therapy combined with neoadjuvant ICI enhances the benefits of neoadjuvant ICI in stage III resectable melanoma. Patients with resectable stage III BRAFV600-mutant melanoma were randomized to neoadjuvant pembrolizumab alone, sequential dabrafenib/trametinib followed by pembrolizumab, or concurrent triple therapy. A pPR was achieved in 55% of patients with pembrolizumab monotherapy, 50% in the sequential therapy arm, and 80% in the concurrent therapy arm. MPR correlated best with durable RFS, particularly in the pembrolizumab monotherapy arm, where no patients with an MPR experienced recurrence. Despite higher response rates with concurrent therapy, toxicity was significantly greater, with 55% of patients experiencing grade 3–4 irAEs and a 40% neoadjuvant treatment discontinuation rate. At 2 years, EFS was 60% with pembrolizumab alone, 80% with sequential therapy, and 71% with concurrent therapy. Although the response rate was highest with concurrent therapy, the quality and duration of response must be observed carefully. Recurrences following pathological response were more common when targeted therapy was incorporated, questioning the durability of response when combining BRAF/MEK inhibitors with immunotherapy in the neoadjuvant setting.

### 4.9. The NIVEC Trial: Neoadjuvant Nivolumab + T-VEC Combination Therapy for Resectable Early-Stage or Metastatic (IIIB-IVM1a) Melanoma with Injectable Disease [22]

Talimogene laherparepvec (T-VEC), a modified herpes simplex virus type-1 (HSV-1), is a local oncolytic viral immunotherapy approved for patients with unresectable stage IIIB-IVM1a. Intralesional injections of T-VEC heightens the immune response by increasing infiltrating CD8^+^ T cells and IFN-γ gene and PD-L1 protein expression within the tumor microenvironment, thus turning an immune-desolate tumor into an immunogenic tumor, making the tumor more susceptible to ICI treatment. Rohan et al. [22] designed this phase II study to investigate the added efficacy of neoadjuvant intralesional T-VEC combined with systemic nivolumab in patients with resectable stage IIIB–IVM1a melanoma. Patients were to receive four courses of T-VEC (up to 4 mL) and three doses of nivolumab (240 mg) every 2 weeks, followed by surgical resection in week nine. Long-term results of the study have not been published yet. However, preliminary results show that this combination achieved an MPR rate of 65% and a 1-year EFS rate of 75%, with an acceptable safety profile, with only 8% having grade 3 irAEs.

**Table 1:** Summary of trials of neoadjuvant immunotherapy in resectable stage III/IV melanoma.

Trial	Population	Design	Intervention	Primary Endpoint	Toxicity	Response	Findings
4.1. Amaria 2018 [12]	Stage III/Oligometastatic Stage IV	Phase II (N = 23)	A. Neoadj IPI (3 mg/kg) + Nivo 1 mg/kg × 1–3 B. Neoadj Nivo 1 mg/kg	PR	Grade 3+ AEs A.73% B. 8%	pCR A. 45% B. 25%	Combination therapy results in higher pCR with significant toxicity
4.2. OpACIN Blank 2018 [15]	Palpable Stage III	Phase Ib (N = 20)	A. Neoadj IPI 3 mg/kg + Nivo 1 mg/kg > Adj IPI + Nivo × 2 B. Adj IPI 3 mg/kg + Nivo 1 mg/kg × 4	Safety, efficacy	Grade 3/4 AEs 90%	PR 78%	Neoadj superiority over adjuvant. High toxicity.
4.3. OpACIN-neo Rozeman 2019 [16]	Palpable Stage III	Phase II (N = 86)	A. IPI 3 mg/kg + Nivo 1 mg/kg × 2 B. IPI 1 mg/kg + Nivo 3 mg/kg × 2 C. IPI 3 mg/kg × 2 > Nivo 3 mg/kg × 2	Safety, PR	Grade 3/4 AEs A. 40% B. 20% C. 50%	Pathologic response: A. 80% B. 77% C. 65%	Group B—high pathologic response and lowest toxicity
4.5. PRADO Reijers 2022 [17]	Stage IIIB-D	Phase II (N = 99)	Neoadj IPI 1 mg/kg + Nivo 3 mg/kg × 2 > ILN excision > A. MPR > Observation B. pPR > TLND C. pNR > TLND + adjuv CRT	Safety, PR, RFS	Grade 3/4 AEs 22%	MPR 61% = TLND omitted in 59/60. pRR 11% pNR 21% 2-year RFS A. 93% B. 64% C. 71%	Supports response-driven personalization of treatment after neoadj IPI + Nivo
4.4. SWOG S1801 2023 [18]	Stage IIIB-IV	Phase II (N = 313)	A. Neoadju Pembro 200 mg × 3, surgery, Adj Pembro × 15 B. Adj Pembro 200 mg × 18	EFS	Grade 3+ AEs A. 12% B. 14%	2-year EFS A. 72% B. 49%	Better EFS with neoadj–adj than adjuv alone
4.6. NADINA Blank 2024 [19]	Macroscopic Stage III	Phae III	A. Neoadj IPI 1 mg/kg + Nivo 3 mg/kg × 2 > Surgery > a. pMR > Observation b. pPR/pNR > if BRAF-mutated DT × 46w, if BRAF wildtype Nivo × 11 B. Surgery > Adj Nivo × 12	EFS (Progression, recurrence, or death)	Grade 3+ AEs A. 29.7% B. 14.7%	MPR 59%, 12-month EFS A. 83% B. 57%	Neoadj followed by response-driven adjuvant therapy superior to surgery followed by adjuvant
4.7. Amaria 2022 [20]	Resectable Stage III/IV	Phase II (N30)	Neoadj nivo 480 mg + relatlimab 180 mg × 2, then surgery, then adj nivo + relatlimab	pCR Safety	AEs: Neoadj: no grade 3+ Adj: 26% grade 3–4	pCR 57%, Any path response 70% 2-year RFS pCR 91% Any path response 92% No response 55%	Comparable response to other neoadjuvant regimens with less toxicity
4.8. NeoTrio Long 2024 [21]	BRAF^V600^ mutant resectable stage III	Phase II (N = 60)	A. Pembro B. DT > surgery followed by Pembro > surgery C. DT + Pembro > Surgery	PR	Grade 3/4 AEs A. 5% B. 25% C. 55%	PR A. 55% B. 50% C. 80% 2-year EFS A. 60% B. 80% C. 71%	Concurrent therapy highest pathological response rate but uncertain durability
4.9. NIVEC Rohan 2022 [22]	Resectable stage IIIB-IVM1a	Phase II (N = 24)	T-VEC × 4 + Nivo (240 mg) × 3 every 2 weeks >> surgery at week 9	MPR	Grade 3 AEs 8%	MPR 65% 1-year EFS 75%	Improved MPR rates and EFS compared to Nivo alone. Limited preliminary results.

Abbreviations: neoadj—neoadjuvant; adj—adjuvant; IPI—Ipilimumab; Nivo—nivolumab; Pembro—pembrolizumab; AEs—adverse events; ILN—index lymph node; CRT—chemoradiation; PR—pathologic response; MPR—major pathologic response; pCR—pathologic complete response; pPR—pathologic partial response; pNR—pathologic no response; TLND—therapeutic lymph node dissection; RFS—recurrence-free survival; EFS—event-free survival.

## 5. Discussion

Recent trials highlight the growing rationale for neoadjuvant immune checkpoint blockade in patients with resectable stage III melanoma, challenging the traditional approach of surgery followed by adjuvant therapy. Adjuvant PD-1 inhibitors such as nivolumab and pembrolizumab demonstrate significant improvements in RFS in long-term follow-up. The KEYNOTE-054 trial found that adjuvant pembrolizumab significantly improved RFS (55.4% vs. 38.3%) and DMFS (60.6% vs. 44.5%) compared to placebo in patients with resected stage III melanoma [25,26]. However, almost 50% of patients still experience recurrence, and long-term OS benefits have not been consistent across studies [11,25]. In addition, the combination of nivolumab plus ipilimumab in the adjuvant setting failed to improve outcomes in the CheckMate 915 trial and led to significant toxicity [27]. These limitations, combined with the biological potential of neoadjuvant therapy, have prompted a paradigm shift in treatment strategies for high-risk melanoma.

One of the principal biological advantages of neoadjuvant immunotherapy lies in the timing of immune activation. ICIs function by reinvigorating tumor-infiltrating lymphocytes, and their efficacy is enhanced when a tumor antigen source is present. Administering IO before tumor resection allows for in situ priming of the immune system, leading to more robust and polyclonal T cell responses. Preclinical models and translational studies have shown that neoadjuvant therapy induces greater expansion of tumor-specific T cell clones than the same agents given in the adjuvant setting in both the lab and clinical studies [15,28].

As noted above, neoadjuvant approaches demonstrate superior outcomes across multiple trials. The SWOG S1801 trial, which became central to the paradigm shift towards melanoma neoadjuvant therapy, demonstrated a clear benefit of neoadjuvant–adjuvant pembrolizumab over adjuvant-only therapy, with a 2-year EFS of 72% versus 49%. Similarly, the phase III NADINA trial utilizing two cycles of neoadjuvant ipilimumab plus nivolumab significantly improved 12-month EFS compared to standard adjuvant nivolumab (83.7% vs. 57.2%). These results reinforce the hypothesis that early systemic immune engagement can improve long-term disease control. While both monotherapy and combination neoadjuvant immunotherapy show favorable results compared to standard-of-care adjuvant therapy alone, combination therapy is associated with higher rates of complete or near-complete pathologic response, leading to more robust RFS [14,18]. As novel combination regimens continue to evolve, the utility of single-agent checkpoint inhibition in the neoadjuvant setting is anticipated to become increasingly limited.

The discovery and use of newer immunomodulatory agents, such as LAG-3, is promising. LAG-3 negatively regulates T-cell proliferation and function and is frequently co-expressed with PD-1 on exhausted T cells in melanoma. Preclinical models showed synergistic antitumor activity when both LAG-3 and PD-1 were blocked [20]. The RELATIVITY-047 trial demonstrated that combining relatlimab with nivolumab improved PFS over PD-1 monotherapy in unresectable melanoma, with better tolerability compared to CTLA-4-based combinations [29]. In the neoadjuvant setting, relatlimab plus nivolumab combination resulted in 70% pathological response (PR) with 2-year RFS of 92% among patients with any PR, similar to the results of other combination therapies [20]. Yet these promising results are based on a small number of patients without long-term outcomes, which limits their ability to replace neoadjuvant Ipilimumab and Nivolumab.

The neoadjuvant setting enables response-based treatment personalization with possible de-escalation of therapy, a key advantage of this approach [17,19]. Trials such as the PRADO trial demonstrated that patients achieving an MPR after neoadjuvant therapy had RFS exceeding 90% and can safely omit extensive TLND or adjuvant therapy. Unprecedentedly high 24-month RFS (93%) and DMFS (94%) were reported in patients with MPR without extensive surgery and the morbidity of long adjuvant therapy [17]. Conversely, patients with partial or no response may benefit from modification and escalation of postoperative therapy. Adjuvant BRAF and MEK inhibitors may be favored over standard-of-care adjuvant IO in patients with BRAF-mutated melanoma without a significant PR. This response-guided strategy optimizes outcomes and improves quality of life while reducing treatment-related morbidity.

Importantly, neoadjuvant IO has become more tolerable with optimized dosing and newer agents. The OpACIN-neo trial identified a lower-toxicity regimen of ipilimumab (1 mg/kg) plus nivolumab (3 mg/kg) that preserved efficacy while reducing grade 3–4 irAEs [16]. This regimen was applied in subsequent trials such as the PRADO and NADINA trials, with a similar and acceptable toxicity profile [17,19]. The relatlimab plus nivolumab combination further improved tolerability [20].

The rationale for neoadjuvant targeted therapy (BRAF and MEK inhibitors) in melanoma centers on achieving rapid reduction in tumor burden, improving resectability, and potentially enhancing long-term outcomes. Targeted therapy with BRAF and MEK inhibitors induces high radiographic and pathological response rates within weeks, minimizing the risk of disease progression before surgery. In the NeoCombi phase II trial, neoadjuvant dabrafenib plus trametinib for resectable stage IIIB–C BRAF^V600^-mutant melanoma demonstrated high response rates, with 86% of patients achieving a radiological response after 12 weeks and 49% achieving a pCR. Despite the high response rate, 2-year RFS was only 43.4% [30]. To date, no studies have compared the long-term efficacy of neoadjuvant IO vs. targeted therapy. Indirect comparison demonstrates the superiority of IO in achieving durable responses. Early investigations of neoadjuvant triple combination therapy with pembrolizumab, dabrafenib, and trametinib have demonstrated high pathological response rates; however, the associated high incidence of early recurrences and significant treatment-related toxicity raise concerns regarding the viability of this approach [21].

An alternative approach in the neoadjuvant setting was investigated with intra-tumoral injections of Talimogene Laherparepvec (T-VEC). A randomized phase II trial, neoadjuvant T-VEC followed by surgery, was compared to upfront surgery alone in patients with resectable stage IIIB–IVM1a melanoma. Neoadjuvant T-VEC significantly improved 2-year RFS (29.5% vs. 16.5%) and OS (88.9% vs. 77.4%) compared to surgery alone. Although 14.5% of patients in the T-VEC arm did not reach surgery due to disease progression, most failures were associated with distant metastasis, suggesting that immediate surgery may not have been curative [31]. Similarly, two clinical trials have demonstrated its efficacy, in combination with ICIs, in unresectable stage IIIB-IV disease, with objective response rates reaching 62% [32,33]. Building on its mechanism of enhancing local tumor lysis and systemic immune activation, T-VEC combined with nivolumab has shown promising results in the neoadjuvant setting. Preliminary results of the NIVEC trial show that this combination achieved an MPR rate of 65% and a 1-year EFS rate of 75%, with an acceptable safety profile, with only 8% having grade 3 irAEs. These findings suggest that neoadjuvant T-VEC, particularly in combination with ICIs, may offer an effective strategy to improve outcomes in resectable melanoma and warrant further investigation of oncolytic virus-based strategies to enhance neoadjuvant immunotherapy [22].

The National Comprehensive Cancer Network (NCCN) [5] incorporated neoadjuvant immunotherapy as the preferred strategy for the management of resectable stage III melanoma. Current guidelines recommend either neoadjuvant anti-PD–1 monotherapy or neoadjuvant combination of anti-PD–1 and anti-CTLA–4 checkpoint blockade as the preferred regimens (category A). Combination regimens incorporating LAG-3 inhibitors are also listed as an option; however, supporting data remain limited. According to NCCN, neoadjuvant BRAF/MEK inhibitors should only be offered to patients with contraindications to immunotherapy.

## 6. Challenges and Future Directions

Neoadjuvant IO has redefined the management of resectable stage III melanoma, demonstrating superior clinical and pathological response rates compared to the standard adjuvant treatment paradigm. However, several limitations hinder widespread adoption. To date, long-term outcomes remain uncertain, as the majority of available data are derived from early-phase (phase I and II) clinical trials involving limited patient populations and relatively short follow-up durations (1- to 2-year RFS rates). Comprehensive 5-year survival and disease control data from these initial neoadjuvant studies are anticipated and will be critical to fully assess the durability and long-term efficacy of this treatment approach.

IrAEs, particularly grade 3–4 toxicities such as colitis, hepatitis, and endocrinopathies, are frequently observed with neoadjuvant ICIs blockade, particularly in combination regimens, and may result in delays or cancelation of planned curative surgery. However, early-phase studies are promising with low rates of both disease progression and severe toxicity precluding definitive surgery, especially in newer combination regimens [20,23].

Notwithstanding high rates of MPR, a subset of patients nonetheless exhibit primary resistance or experience disease progression during neoadjuvant therapy, leading to loss of resectability and negatively impacting survival outcomes. To date, there are no reliable clinical variables effectively predicting response to neoadjuvant IO. Identifying patients who will benefit from neoadjuvant IO remains of critical importance to avoid unnecessary, expensive and morbid therapy. High IFN-γ gene expression signature and high TMB along with high pCR rate may play a more significant role in predicting response to immunotherapy in the future.

Future directions in melanoma therapy are focused on overcoming resistance to current IO and enhancing antitumor immune responses. Novel strategies under investigation include targeting additional ICIs such as LAG-3, TIGIT, and TIM-3, as well as activating innate immune pathways like the cGAS–STING axis. Pharmacological agents stimulating pattern recognition receptors, oncolytic virus therapies (T-VEC), and combinations of ICIs with innate immune activators are being explored to transform the tumor microenvironment and promote durable responses. Furthermore, leveraging microbiome modulation, adoptive cell therapies, and novel adjuvant and neoadjuvant approaches aim to extend survival benefits even in patients who fail first-line ICIs [2,31,34,35].

## 7. Conclusions

Neoadjuvant IO fundamentally redefined the treatment paradigm for patients with resectable stage III melanoma. By initiating systemic immune activation prior to surgery, neoadjuvant approaches leverage the presence of tumor antigens to generate more robust and durable antitumor responses compared to traditional adjuvant therapy. Clinical trials consistently demonstrate that achieving an MPR strongly correlates with improved RFS and OS, supporting the integration of PR as a critical prognostic marker and decision-making tool. Optimized combination regimens and response-adapted strategies have further improved the balance between efficacy and toxicity, allowing treatment personalization that minimizes morbidity without compromising outcomes. While challenges such as immune-related toxicity, disease progression during therapy, and biomarker-driven patient selection remain, emerging agents and novel immunomodulatory targets promise to further refine and enhance neoadjuvant strategies. As longer-term data mature, neoadjuvant IO is emerging as a leading strategy for high-risk, resectable melanoma, offering patients the opportunity for greater responses, reduced treatment toxicity, and improved long-term survival.

## References

[1] Trimble E.L., Ungerleider R.S., Abrams J.A., Kaplan R.S., Feigal E.G., Smith M.A., Carter C.L., Friedman M.A. (1993). Neoadjuvant therapy in cancer treatment. Cancer.

[2] Knight A., Karapetyan L., Kirkwood J.M. (2023). Immunotherapy in Melanoma: Recent Advances and Future Directions. Cancers.

[3] Robert C., Schachter J., Long G.V., Arance A., Grob J.J., Mortier L., Daud A., Carlino M.S., McNeil C., Lotem M. (2015). Pembrolizumab versus Ipilimumab in Advanced Melanoma. N. Engl. J. Med..

[4] Wolchok J.D., Chiarion-Sileni V., Rutkowski P., Cowey C.L., Schadendorf D., Wagstaff J., Queirolo P., Dummer R., Butler M.O., Hill A.G. (2025). Final, 10-Year Outcomes with Nivolumab plus Ipilimumab in Advanced Melanoma. N. Engl. J. Med..

[5] Network N.C.C. Melanoma (Version 2.2025). https://www.nccn.org/professionals/physician_gls/pdf/cutaneous_melanoma.pdf.

[6] Bray F., Laversanne M., Sung H., Ferlay J., Siegel R.L., Soerjomataram I., Jemal A. (2024). Global cancer statistics 2022: GLOBOCAN estimates of incidence and mortality worldwide for 36 cancers in 185 countries. CA A Cancer J. Clin..

[7] Siegel R.L., Miller K.D., Fuchs H.E., Jemal A. (2022). Cancer statistics, 2022. CA A Cancer J. Clin..

[8] Hamid O., Robert C., Daud A., Hodi F.S., Hwu W.J., Kefford R., Wolchok J.D., Hersey P., Joseph R., Weber J.S. (2019). Five-year survival outcomes for patients with advanced melanoma treated with pembrolizumab in KEYNOTE-001. Ann. Oncol. Off. J. Eur. Soc. Med. Oncol..

[9] Larkin J., Chiarion-Sileni V., Gonzalez R., Grob J.-J., Rutkowski P., Lao C.D., Cowey C.L., Schadendorf D., Wagstaff J., Dummer R. (2019). Five-Year Survival with Combined Nivolumab and Ipilimumab in Advanced Melanoma. N. Engl. J. Med..

[10] Eggermont A.M.M., Blank C.U., Mandalà M., Long G.V., Atkinson V., Dalle S., Haydon A., Lichinitser M., Khattak A., Carlino M.S. (2018). Adjuvant Pembrolizumab versus Placebo in Resected Stage III Melanoma. N. Engl. J. Med..

[11] Larkin J., Del Vecchio M., Mandalá M., Gogas H., Fernandez A.M.A., Dalle S., Cowey C.L., Schenker M., Grob J.-J., Chiarion-Sileni V. (2023). Adjuvant Nivolumab versus Ipilimumab in Resected Stage III/IV Melanoma: 5-Year Efficacy and Biomarker Results from CheckMate 238. Clin. Cancer Res. Off. J. Am. Assoc. Cancer Res..

[12] Amaria R.N., Reddy S.M., Tawbi H.A., Davies M.A., Ross M.I., Glitza I.C., Cormier J.N., Lewis C., Hwu W.-J., Hanna E. (2018). Neoadjuvant immune checkpoint blockade in high-risk resectable melanoma. Nat. Med..

[13] Rozeman E.A., Hoefsmit E.P., Reijers I.L.M., Saw R.P.M., Versluis J.M., Krijgsman O., Dimitriadis P., Sikorska K., van de Wiel B.A., Eriksson H. (2021). Survival and biomarker analyses from the OpACIN-neo and OpACIN neoadjuvant immunotherapy trials in stage III melanoma. Nat. Med..

[14] Menzies A.M., Amaria R.N., Rozeman E.A., Huang A.C., Tetzlaff M.T., van de Wiel B.A., Lo S., Tarhini A.A., Burton E.M., Pennington T.E. (2021). Pathological response and survival with neoadjuvant therapy in melanoma: A pooled analysis from the International Neoadjuvant Melanoma Consortium (INMC). Nat. Med..

[15] Blank C.U., Rozeman E.A., Fanchi L.F., Sikorska K., van de Wiel B., Kvistborg P., Krijgsman O., van den Braber M., Philips D., Broeks A. (2018). Neoadjuvant versus adjuvant ipilimumab plus nivolumab in macroscopic stage III melanoma. Nat. Med..

[16] Rozeman E.A., Menzies A.M., van Akkooi A.C.J., Adhikari C., Bierman C., van de Wiel B.A., Scolyer R.A., Krijgsman O., Sikorska K., Eriksson H. (2019). Identification of the optimal combination dosing schedule of neoadjuvant ipilimumab plus nivolumab in macroscopic stage III melanoma (OpACIN-neo): A multicentre, phase 2, randomised, controlled trial. Lancet Oncol..

[17] Reijers I.L.M., Menzies A.M., van Akkooi A.C.J., Versluis J.M., van den Heuvel N.M.J., Saw R.P.M., Pennington T.E., Kapiteijn E., van der Veldt A.A.M., Suijkerbuijk K.P.M. (2022). Personalized response-directed surgery and adjuvant therapy after neoadjuvant ipilimumab and nivolumab in high-risk stage III melanoma: The PRADO trial. Nat. Med..

[18] Patel S.P., Othus M., Chen Y., Wright G.P., Yost K.J., Hyngstrom J.R., Hu-Lieskovan S., Lao C.D., Fecher L.A., Truong T.-G. (2023). Neoadjuvant–Adjuvant or Adjuvant-Only Pembrolizumab in Advanced Melanoma. N. Engl. J. Med..

[19] Blank C.U., Lucas M.W., Scolyer R.A., van de Wiel B.A., Menzies A.M., Lopez-Yurda M., Hoeijmakers L.L., Saw R.P.M., Lijnsvelt J.M., Maher N.G. (2024). Neoadjuvant Nivolumab and Ipilimumab in Resectable Stage III Melanoma. N. Engl. J. Med..

[20] Amaria R.N., Postow M., Burton E.M., Tetzlaff M.T., Ross M.I., Torres-Cabala C., Glitza I.C., Duan F., Milton D.R., Busam K. (2022). Neoadjuvant relatlimab and nivolumab in resectable melanoma. Nature.

[21] Long G.V., Carlino M.S., Au-Yeung G., Spillane A.J., Shannon K.F., Gyorki D.E., Hsiao E., Kapoor R., Thompson J.R., Batula I. (2024). Neoadjuvant pembrolizumab, dabrafenib and trametinib in BRAFV600-mutant resectable melanoma: The randomized phase 2 NeoTrio trial. Nat. Med..

[22] Rohaan M.W., Stahlie E.H.A., Franke V., Zijlker L.P., Wilgenhof S., van der Noort V., van Akkooi A.C.J., Haanen J.B.A.G. (2022). Neoadjuvant nivolumab + T-VEC combination therapy for resectable early stage or metastatic (IIIB-IVM1a) melanoma with injectable disease: Study protocol of the NIVEC trial. BMC Cancer..

[23] Moody J., Botham S., Dahill K., Wallace D., Hardwicke J. (2017). Complications following completion lymphadenectomy versus therapeutic lymphadenectomy for melanoma—A systematic review of the literature. Eur. J. Surg. Oncol. J. Eur. Soc. Surg. Oncol. Br. Assoc. Surg. Oncol..

[24] Menzies A.M., Saw R.P.M., Lo S.N., Gonzalez M., Ch’ng S., Nieweg O.E., Shannon K.F., Ferguson P.M., Lee J.H., Rizos H. (2022). Neoadjuvant dabrafenib and trametinib (D+T) for stage III melanoma: Long-term results from the NeoCombi trial. J. Clin. Oncol..

[25] Eggermont A.M.M., Kicinski M., Blank C.U., Mandala M., Long G.V., Atkinson V., Dalle S., Haydon A., Meshcheryakov A., Khattak A. (2022). Five-Year Analysis of Adjuvant Pembrolizumab or Placebo in Stage III Melanoma. NEJM Evid..

[26] Wolchok J.D., Chiarion-Sileni V., Gonzalez R., Rutkowski P., Grob J.-J., Cowey C.L., Lao C.D., Wagstaff J., Schadendorf D., Ferrucci P.F. (2017). Overall Survival with Combined Nivolumab and Ipilimumab in Advanced Melanoma. N. Engl. J. Med..

[27] Weber J.S., Schadendorf D., Vecchio M.D., Larkin J., Atkinson V., Schenker M., Pigozzo J., Gogas H., Dalle S., Meyer N. (2023). Adjuvant Therapy of Nivolumab Combined With Ipilimumab Versus Nivolumab Alone in Patients With Resected Stage IIIB-D or Stage IV Melanoma (CheckMate 915). J. Clin. Oncol..

[28] Oba T., Kajihara R., Yokoi T., Repasky E.A., Ito F. (2021). Neoadjuvant in situ immunomodulation enhances systemic antitumor immunity against highly metastatic tumors. Cancer Res..

[29] Tawbi H.A., Schadendorf D., Lipson E.J., Ascierto P.A., Matamala L., Gutiérrez E.C., Rutkowski P., Gogas H.J., Lao C.D., Menezes J.J.D. (2022). Relatlimab and Nivolumab versus Nivolumab in Untreated Advanced Melanoma. N. Engl. J. Med..

[30] Long G.V., Saw R.P.M., Lo S., Nieweg O.E., Shannon K.F., Gonzalez M., Guminski A., Lee J.H., Lee H., Ferguson P.M. (2019). Neoadjuvant dabrafenib combined with trametinib for resectable, stage IIIB–C, BRAFV600 mutation-positive melanoma (NeoCombi): A single-arm, open-label, single-centre, phase 2 trial. Lancet Oncol..

[31] Dummer R., Gyorki D.E., Hyngstrom J.R., Ning M., Lawrence T., Ross M.I. (2023). Final Results of Neoadjuvant T-VEC Plus Surgery in Advanced Melanoma. JAMA Oncol..

[32] Ribas A., Dummer R., Puzanov I., VanderWalde A., Andtbacka R.H.I., Michielin O., Olszanski A.J., Malvehy J., Cebon J., Fernandez E. (2017). Oncolytic Virotherapy Promotes Intratumoral T Cell Infiltration and Improves Anti-PD-1 Immunotherapy. Cell.

[33] Chesney J., Puzanov I., Collichio F., Singh P., Milhem M.M., Glaspy J., Hamid O., Ross M., Friedlander P., Garbe C. (2018). Randomized, Open-Label Phase II Study Evaluating the Efficacy and Safety of Talimogene Laherparepvec in Combination With Ipilimumab Versus Ipilimumab Alone in Patients With Advanced, Unresectable Melanoma. J. Clin. Oncol. Off. J. Am. Soc. Clin. Oncol..

[34] Garland K.M., Rosch J.C., Carson C.S., Wang-Bishop L., Hanna A., Sevimli S., Van Kaer C., Balko J.M., Ascano M., Wilson J.T. (2021). Pharmacological Activation of cGAS for Cancer Immunotherapy. Front. Immunol..

[35] Gan Y., Li X., Han S., Liang Q., Ma X., Rong P., Wang W., Li W. (2022). The cGAS/STING Pathway: A Novel Target for Cancer Therapy. Front. Immunol..

